# Developing Adventitious Root Meristems Induced by Layering for Plant Chromosome Preparation

**DOI:** 10.3390/ijms252111723

**Published:** 2024-10-31

**Authors:** Xu Yan, Zizhou Wu, Honglin Wang, Yanchun Zuo, Zhouhe Du

**Affiliations:** 1 Institute of Special Economic Animals and Plants, Sichuan Academy of Agricultural Sciences, Nanchong 637000, China; wuzizhou@scsaas.cn (Z.W.); hlwang@scsaas.cn (H.W.); zuoyanchun@scsaas.cn (Y.Z.); 2 Forage Crops Germplasm Innovation and Production Management Key Laboratory of Nanchong City, Sericulture Research Institute, Sichuan Academy of Agricultural Sciences, Nanchong 637000, China

**Keywords:** layering, adventitious root, plant chromosome preparation, *Zea*, *Brassica*

## Abstract

Chromosome numbers and morphology are important characteristics of a species and its evolution. Root tips are the most commonly used tissue as a source of actively dividing cells for chromosome visualization in plants. Previously, rapidly growing root tips were collected from germinating kernels or from seedlings growing in pots or fields. However, the use of adventitious roots (ARs) derived from aerial tissue as meristems for chromosome visualization has always been overlooked. Here, we successfully induced ARs in 12 materials that were investigated, with the exception of *Sorghum nitidum*. Using ARs meristem we obtained high-quality chromosome spreads for *Morus alba*, *Broussonetia papyrifera*, *Lolium multiflorum*, *Sorghum sudanense*, *S*. *propinquum*, *S*. *bicolor* × *S*. *sudanense*, *Zea mays*, *Z*. *mexicana*, *Glycine max*, *Medicago sativa*, and *Brassica napus*. The results reported here demonstrate that layering is an alternative and effective method for producing meristematic cells for high-quality chromosome preparation in plant species producing ARs. For species that produce ARs by layering, this protocol is particularly valuable for the development of cost-effective and high-throughput non-invasive cytogenetic studies.

## 1. Introduction

Knowledge of chromosome number and morphological features in plants is of great importance in systematics, phylogeny, taxonomy, and plant breeding, especially for aneuploidy detection, ploidy determination, and detection of largescale structural alterations [1]. The cornerstone of the approach for recording chromosome number and morphological features is chromosome preparation. Metaphase chromosomes are typically used for chromosomal analysis because they are highly condensed and can be easily identified using a light microscope [2]. Three main methods have been developed for chromosome preparations, including squashing, spreading, and dropping [2]. More recently, a novel method named ‘SteamDrop’ was also developed, which uses water steam for the preparation of well-spread and morphologically intact chromosomes [3]. Of these methods, the squashing method is the most commonly used technique for chromosome analysis. Moreover, many modern molecular cytogenetic techniques, such as fluorescence in situ hybridization (FISH), fiber FISH, Tyramide-FISH, and genomic in situ hybridization (GISH), require chromosome preparation through spreading or dropping.

Plant chromosome preparations have typically relied on tissues that contain actively dividing cells [4]. A tissue containing a high proportion of dividing cells is the most suitable starting point for subsequent cytogenetic analyses. Fast-growing meristematic tissues, such as root tips, shoot tips, leaf tips, tendril tips, flower buds, anthers, pistils, corollas, protoplasts, or calli from tissue culture, serve as sources of meristem for metaphase chromosome preparations [2,4,5,6]. Among these, root tips are most commonly chosen as a source of meristem material because the mitotic index is generally low in other tissues [2,7]. Usually, other meristems are only considered when the root tip cannot be obtained. For instance, young leaf tissues (shoot-tips) were used to obtain metaphase chromosomes because it is not possible to obtain root tips from mature tree plants in the field, and cuttings do not readily develop roots in birch (*Betula* L.) tree species [8]. Meanwhile, for certain plants like the Gramineae family, the root tips seem to be the only source of meristem for metaphase chromosome preparations, excluding cells from tissue culture.

Root tips can be obtained from germinating seeds at short notice or from actively growing plants at an early developmental stage [6]. However, high-quality root tips are not always available [4]. For example, sometimes seeds are difficult to germinate, root development is weak, or mature individuals have thin roots [7]. Moreover, not all root tips yield a sufficient number of mitotic chromosome spreads (typically, 20% of seeds yield high numbers) [1]. In some studies, it is often necessary to conduct cytogenetic analysis on individual plants. The number of root tips produced by a germinated seed is limited, and the plant faces the risk of premature death after the removal of root tips. There is a need to provide a sufficient number of root tips for chromosome preparation. A method for acquiring root tips from potted young plants was widely used. However, this method is very time-consuming, laborious, tedious, and expensive, particularly when dealing with large-scale materials. Whether root tips are excised from seeds or seedlings, the subsequent growth and development of the plant are affected. Therefore, developing a simple, convenient, and non-destructive method to obtain a large number of root tips for chromosome preparation undoubtedly helps advance the research field of plant cytogenetics.

In any case, actively growing meristematic tissues (tissues containing actively dividing cells) are a prerequisite for preparing chromosome spreads and subsequent cytogenetic analyses. For plant chromosome preparation, any tissue containing dividing cells can be used [6]. In terms of the source of root meristem tissue for plant chromosome preparation, root tips from actively growing plants are always sourced from primary or lateral roots or from adventitious roots beneath the soil layer. Adventitious roots (ARs) are roots that form from aerial tissues derived from stems or leaves. ARs formation is a complex process whose organogenesis is attributed to a crosstalk between endogenous and environmental factors [9]. Many plant species can spontaneously develop ARs to provide additional anchorage and support, enhance water and nutrient capture, or to assist in the survival of a species, or for the propagation of new plant material for the growth of new plants from the parent plant [9]. In some cases, it can be induced by abiotic stresses, such as nutrient deficiency, waterlogging, and physical injury. In horticulture, agriculture, and forestry, cuttings and air layering are widely used to clone plants through the ARs formed by wounding, the latter being usually applied in difficult-to-root plant species. Air layering, a traditionally asexual propagation technique first used in China over 4000 years ago [10], is a simple vegetative technique characterized by the initiation of ARs on one part of the parent plant in situ [11]. This method offers a higher rooting rate than cuttings, genetic uniformity, rapid field establishment, and the ability to reach maturity in a shorter time. ARs beneath the soil layer have long been used for plant chromosome preparation, as demonstrated in *Aegilops mutica* [12], sugarcane [13], and other asexual crops [14,15]. However, to the best of our knowledge, ARs from air layering (or other layering methods) have not been used for chromosome preparation since they were used during early plant research via microscopy.

Even today, root tips of ARs, induced by layering, are still neglected as a material source for chromosome preparation. Here, the layering method was applied to 13 plant materials from 8 genera, including 7 moncotyledonous species and 6 dicotyledonousspecies. ARs of 12 of the studied materials were successfully induced, except for one-year-old *Sorghum nitidum*. Subsequently, we demonstrate that ARs induced by layering forms an excellent tissue source for cytological work using conventional squash-spread techniques.

## 2. Results

### 2.1. Adventitious Root Induction by Layering

**Figure 1:**
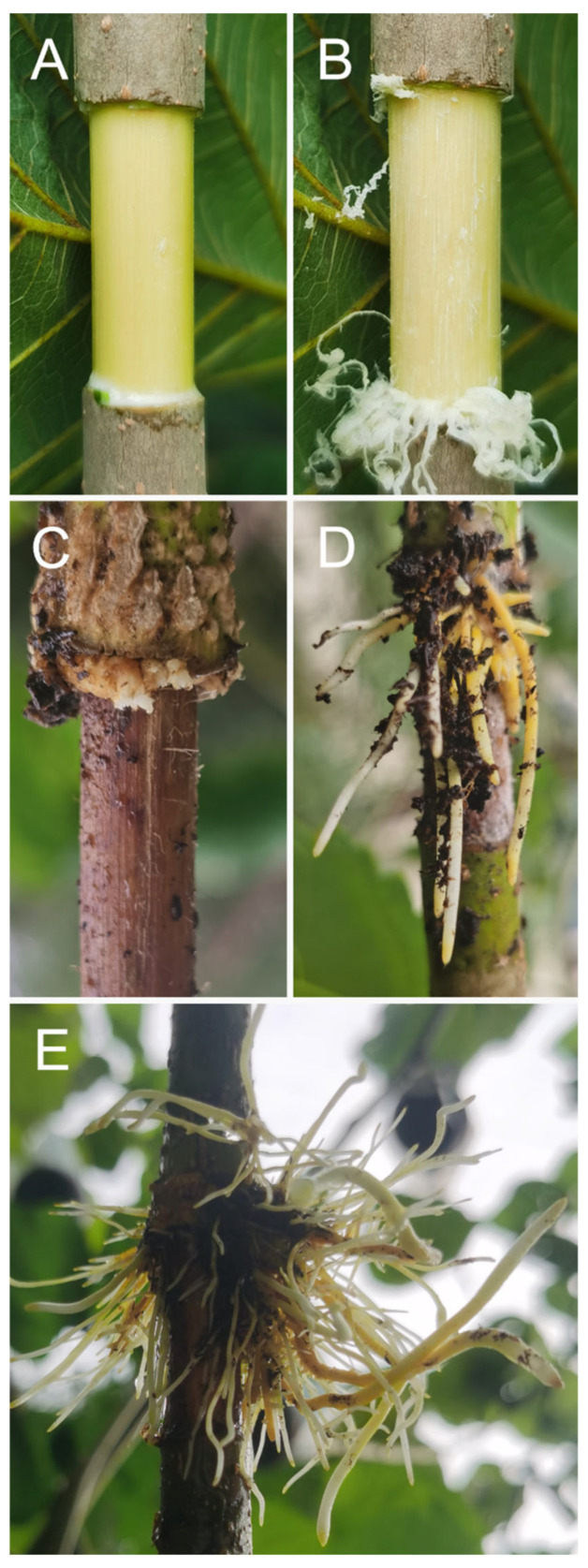
Induction of ARs by air layering in *Morus alba* ‘Da10’. (**A**) Two-centimeter ring-stripping incision. (**B**) Removal of the cambium layer. (**C**) Callus of air layering shoot at 15 days after air layering. (**D**) ARs under air layering at 30 days after air layering. (**E**) ARs under air layering at 45 days after air layering.

**Figure 2:**
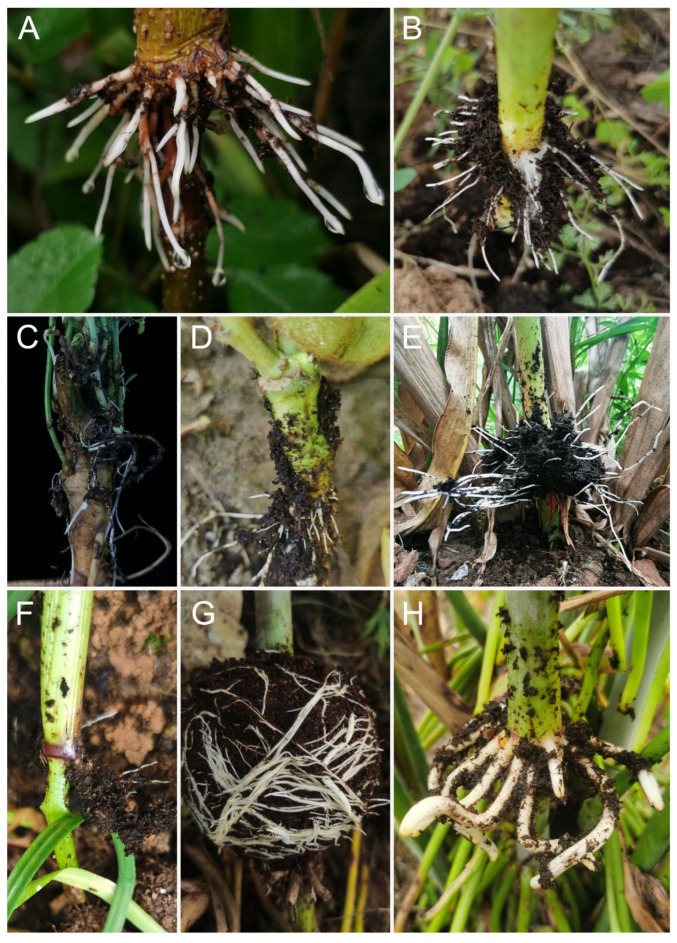
ARs induced by layering. The species (days after layering in parentheses) include *Broussonetia papyrifera* (31 d) (**A**), *Brassica napus* (27 d) (**B**), *Medicago sativa* (18 d) (**C**), *Glycine max* (15 d) (**D**), *Zea mexicana* (8 d) (**E**), *Lolium multiflorum* (15 d) (**F**), *Sorghum propinquum* (7 d) (**G**), and *S*. *bicolor* × *S*. *sudanense* (5 d) (**H**).

Among the materials studied, only one-year-old *Sorghum nitidum* did not induce aerial ARs (Supplementary Figure S1). *Morus alba* (mulberry) undergoes callus formation and adventitious root formation after air layering (Figure 1). Callus formation was observed 15 days after air layering in air-layered branches of mulberry, and several roots were observed 30 days after air layering (Figure 1C,D). The initial roots were very thick. After obtaining the root tips, additional and thinner roots developed from callus tissue 45 days after air layering (Figure 1E). In this example, 85 root tips (>0.5 mm) were collected at 30 and 45 days after air layering. The rooting process of *Broussonetia papyrifera* (paper mulberry) is similar to that of mulberry (Figure 2A). We also induced ARs from aboveground stems of *Brassica napus* (rapeseed) (Figure 2B) and *Glycine max* (soybean) (Figure 2D) through air layering. Meanwhile, for rapeseed and soybeans, mature seeds can still be harvested after air layering. Moreover, *Medicago sativa* (alfalfa) produces ARs using mound layering (Figure 2C).

**Figure 3:**
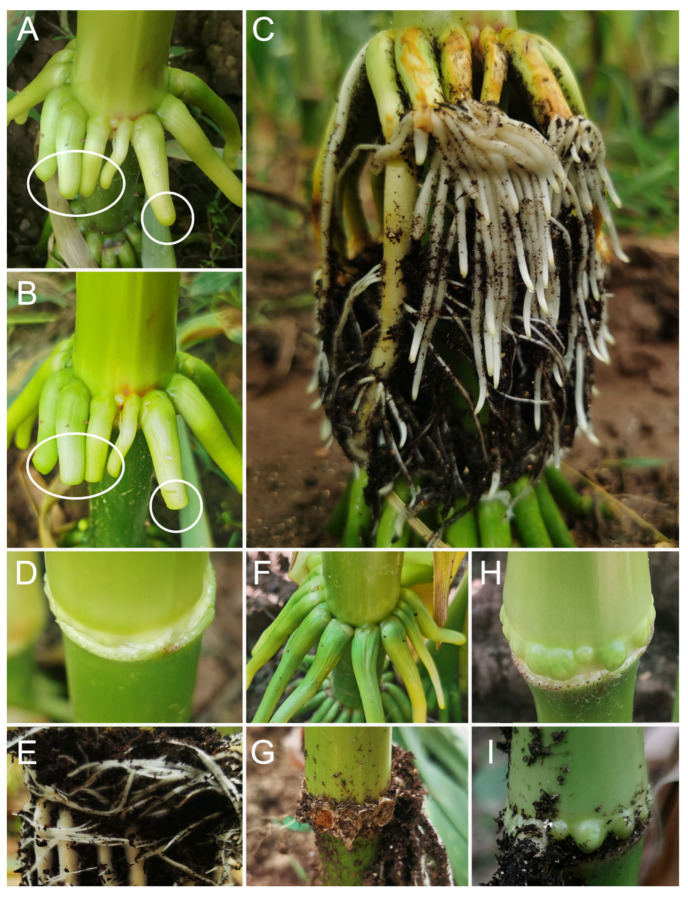
ARs induced by mound layering in maize. (**A**–**C**) After removal of the root cap of the crown roots, ARs sprouted from the layered crown roots (8 days after mound layering in Panel C). (**D**,**E**) During tasseling, the leaf of a basal node was removed, then ARs sprouted from the layered basal node (8 days after mound layering in Panel E). (**F**,**G**) After the removal of the crown roots, a few ARs sprouted from the layered basal node. (**H,I**) Mound layering was performed at 8 days after silking, then a few ARs sprouted from the layered basal node.

We employed four strategies to induce the ARs at maize stem nodes (Figure 3). After removing the root caps of the newly formed aerial roots, we stimulated the growth of 156 secondary ARs by mound layering (Figure 3A–C and Figure S2). The crown roots that have been cut off from the root cap area still retain meristematic tissue at their ends, leading to the ARs formation after layering. Stem nodes that do not produce ARs during flowering can still produce ARs through mound layering (Figure 3D,E). However, this process is so rapid that we missed the optimal time (maybe 3–4 days after layering) to collect the root tips of ARs. After the crown roots are removed from the layered basal node, only a few ARs can sprout from the previously unrooted area of the stem node (Figure 3F,G). The nodes were layered at 8 days after silking, then a few ARs were observed from the layered basal nodes 8 days after mound layering (Figure 3H,I). This indicates that the timing of layering is crucial, and if the timing is missed, the stem nodes will weaken or lose their ability to differentiate into ARs. It is likely that the cells are too differentiated to reverse their program of development to form ARs. Stem nodes of *Zea mexicana* (Mexican teosinte) during flowering that has not yet grown ARs also produce abundant ARs through mound layering (Figure 2E). Sorghum (except *Sorghum nitidum*) (Figure 2G,H) and *Lolium multiflorum* (Italian ryegrass) plants (Figure 2F) have also shown successful rooting through mound layering during flowering. In summary, for Gramineae plants, the formation of ARs may be induced by mound layering during flowering.

### 2.2. Preparation of Chromosomes from ARs Meristems

**Figure 4:**
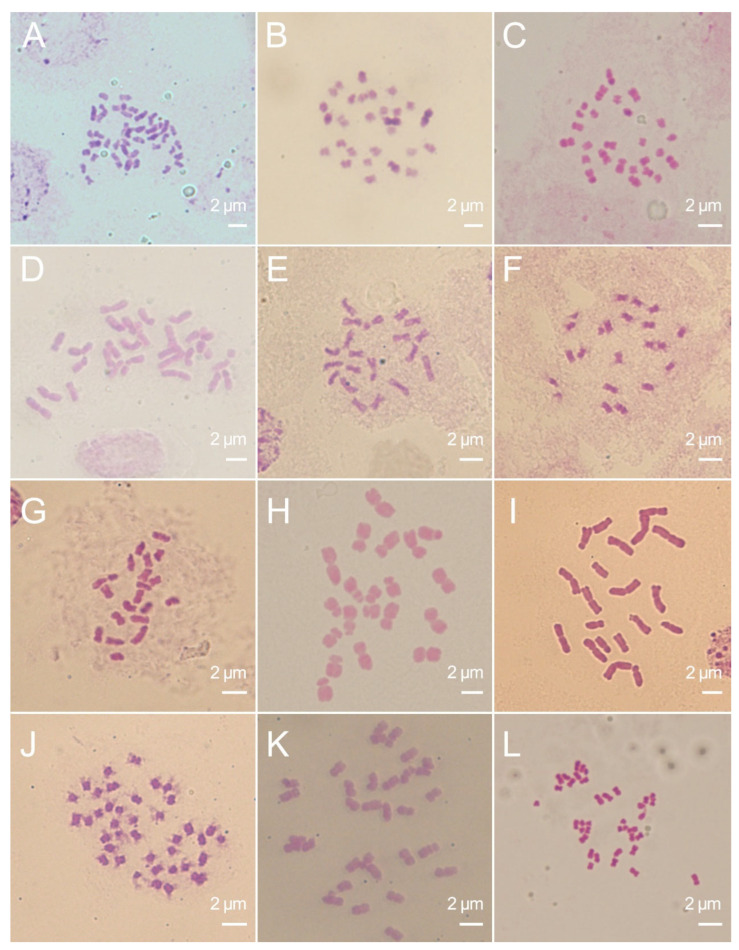
Mitotic metaphase spread from the root tip cells of ARs using the squash method. The species (chromosome number in parentheses) include *Morus alba* ‘Da10’(2*n* = 42) (**A**), *M*. *alba* ‘Heyebai’ (2*n* = 28) (**B**), *Broussonetia papyrifera* (2*n* = 26) (**C**), *Lolium multiflorum* (2*n* = 28) (**D**), *Sorghum sudanense* (2*n* = 20) (**E**), *S*. *propinquum* (2*n* = 20) (**F**), *S*. *bicolor* × *S*. *sudanense* (2*n* = 20) (**G**), *Zea mays* (2*n* = 20) (**H**), *Z*. *mexicana* (2*n* = 20) (**I**), *Glycine max* (2*n* = 40) (**J**), *Medicago sativa* (2*n* = 32) (**K**), and *Brassica napus* (2*n* = 38) (**L**).

We evaluated the potential of ARs as a source for chromosome preparation. As expected, it is feasible to use aerial tissue-derived ARs for chromosome preparation (Figure 4). For example, mulberry chromosomes made from ARs are clearer than those from stem tips. In the case of maize, based on our experience, the metaphase index obtained through ARs is consistent with that obtained using seed root tips. Meanwhile, the chromosome morphology produced by this method is comparable to the chromosomes previously produced using maize seed root tips. This allows for karyotype analysis based on chromosome arm ratio (Supplementary Figure S3). These results demonstrate that root tips of ARs can be an excellent tissue source for chromosome preparation.

## 3. Discussion

### 3.1. Applications of the Method

The protocol detailed here yields chromosome preparations that are suitable for chromosome counting and ploidy identification. Moreover, the combination of this chromosome preparation from ARs with in situ hybridization (ISH) of DNA probes (oligo-FISH, FISH, and GISH) could be widely applied to explore genome organization in plants. This technique can be used for various purposes such as karyotyping, chromosomal mapping, phylogenetic, and taxonomic studies, and for integrating physical, genetic, and cytogenetic maps.

The protocol is theoretically applicable to any species producing ARs. Most plants can be air layered. For example, if a plant is capable of producing aerial roots naturally, it can be air layered. Similarly, if a plant can be propagated through prostrate stems or cuttings from culms, rhizomes, and branches, it can also be rooted through air layering. To date, many important crops, fruits, and endangered plants have been propagated by air layering (Supplementary Table S1), including 104 species from 79 genera and 47 families.

Moreover, some plants can produce ARs from cuttings or through tip layering, simple layering, compound layering, or mound (stool) layering (Supplementary Figure S4). ARs induced by aeroponics were reported in *Brassica* species [16]. ARs can also be induced by tissue culture or in hydroponic systems. ARs of some plants were induced by tissue culture, as reported in apple [17], bambara groundnut [*Vigna subterranea* (L.) Verdc.] [18], and *Cuphea aequipetala* Cav. [19]. ARs of some plants were induced by hydroponic cultivation, as reported in poplar (*Populus*) [20], *Prunus* [21], and Danti (*Baliospermum montanum* L.) [22]. The advantage of both methods is that ARs can be generated regardless of the season. ARs of most succulent forms from the leaf, such as begonia (*Begonia* × *tuberhybrida* Voss) [23], African violet (*Saintpaulia ionantha*) [24], and ZZ plant (*Zamioculcas zamiifolia*) [25]. These are also potential methods for obtaining the ARs meristems for chromosome preparation.

In fact, the underground root tips obtained by potted plants also develop from stems. Taking maize as an example, the lateral terminal roots of the germinating seed are developed from the first stem node (Supplementary Figure S2). We obtained root tips from potted mulberry plants, and young root tips which were suitable for chromosome analysis also originated from the basal stem (Supplementary Figure S5). In short, aboveground ARs, like belowground root tips, can be used for chromosome research.

### 3.2. Comparison to Other Methods

Root tips are the most commonly used meristems for chromosome preparation. However, germinating seeds often have a primary root, and in some plants, seeds may be scarce or non-existent. Meanwhile, not all root tips (typically 20% of seeds) yield a sufficient number of mitotic chromosome spreads [1]. Pure lines and single crosses with an identical genetic background have sufficient seed roots for chromosome preparation. However, the chromosome information of seeds produced by heterozygous parents may vary. Obtaining root tips through seed germination carries the risk of growth delay or death after cutting the root tips. Therefore, taking seed root is no longer suitable for these materials.

Taking root tips from potted plants involves soil preparation, potting, transplanting seedlings, and plant care [15]. Frequent watering is required for the potted plant. When harvesting roots, the entire plant in the pot needs to be lifted and placed back, and the root tips from the soil surface are taken. This process can be labor-intensive, especially when dealing with large quantities of plant lines or replicates. Frequent lifting and repotting of the plant can affect plant growth and yield formation. Young root tips that come into contact with the inner wall of the pot are susceptible to burning during high summer temperatures. High-quality root tips that are hidden in the soil are not easily obtained. In order to obtain them, it is necessary to remove the soil and expose the root system, which can significantly impede plant growth. Obviously, studying the agronomic traits of potted plants is not appropriate.

The use of ARs as meristems for chromosome preparation has always been overlooked, and this study confirms their effectiveness in chromosome preparation. This method only requires a small amount of sphagnum moss, a box, and black polyethylene film, reducing the material cost needed to obtain root tips compared to the potted method. Obtaining ARs from the aboveground parts of plants reduces labor intensity. The growth of the underground roots of the plant is not disturbed, enabling an accurate assessment of the growth potential and stress status of the plant. For woody plants, the air layers were cut from the mother plant and then planted for further research. For Gramineae plants, potted plants can generally obtain root tips after at least 30 days, while this method can induce rooting after approximately 1 week. In terms of obtaining the number of root tips, this method can obtain at least 5 or more young root tips and up to more than 100 young root tips for chromosome preparation (Supplementary Figure S5). Moreover, this method allows for many rounds of root tip collection. Layering is a low-cost and simple method that is easy for researchers to use. Researchers are not faced with the challenges of insufficient quantities or poor quality of root tips, which could cause delays or failures in experimental analyses. As noted by Anamthawat-Jónsson [8], “Cytogenetics cannot be applied in population studies unless samples are obtained from the actual plants in the field”. The protocol for chromosome preparation described here is highly effective for studying cytogenetics and population genetics of plants in their natural habitat, such as *Zea* and *Brassica*.

Compared to the shoot-tip chromosomes of mulberry, ARs chromosomes tend to be shorter or more condensed, which is beneficial for chromosome counting (Supplementary Figure S6). This finding is similar to the results of a previous study on the shoot-tip and root-tip chromosomes of birch [8]. The chromosome morphology of the ARs and seminal roots is similar in maize (Supplementary Figure S6). These results indicate that ARs are reliable meristem tissues for chromosome research.

### 3.3. Limitations of the Method

ARs may not always be accessible, depending on weather conditions and general plant physiology. Rooting success also depends on the plant species and genotype. This method is incapable for species that fail to produce ARs. For example, the one-year-old *Sorghum nitidum* was not successfully induced (Supplementary Figure S1). Some woody plants may take a long time to initiate ARs through air layering. In *Eusideroxylon zwageri*, it takes approximately six to eight months [26]. Additionally, it is not feasible to stay in a forest to do ARs and collect root tips for chromosome study.

## 4. Materials and Methods

### 4.1. Plant Materials

The materials used in this study included two types of plants (woody and herbaceous), four families, eight genera, and thirteen materials (Table 1).

### 4.2. Root Preparations

Layering was carried out in Shunqing Nanchong (30°86′ N, 106°06′ E) in August 2022 (*Morus* and *Broussonetia*), in September 2022 (*Sorghum*, *Zea mexicana*, and *Glycine*), in October 2022 (*Medicago*), in March 2023 (*Lolium* and *Brassica*), and in July 2023 (*Z. mays*). Herbaceous plants were treated during the flowering period. Three stems from each species were used to induce ARs.

For *Sorghum*, *Zea*, *Lolium*, and *Medicago* plants, boxes with holes (Supplementary Figure S7) were used to directly wrap a basal stem node (Gramineae) or crown (*Medicago*) of the plant. For the other species investigated, before using the boxes for wrapping stems at the place of bark removal, about a 2 cm ring of the phloem and the cambium layer was removed by making two parallel cuts and by joining those cuts with a sharp knife (Supplementary Figure S8A). The boxes, filled with moist PINDSTRUP Substrate (substrate with medium fertilizer content, 100% blonde peat, pH 6.0, 0–6 mm), were then covered with a black polythene sheet and securely tied (Supplementary Figure S8B–D). The air layers were kept moist by regularly spraying them with water once every 7–10 days, and they were continuously monitored for signs of root development. Roots were observed after removing the medium and rinsing them with tap water. Root tips resembling the primary root from seeds were collected during the morning hours (between 9:00 am and 11:00 am). Only root tips of 1–2 cm in length were excised.

### 4.3. Chromosome Preparations

Mitotic chromosome spreads were prepared from ARs meristems using the squashing method (Supplementary Figure S9). In brief, root tips from ARs were pretreated in the dark with metaphase-arresting agents, including *α*-bromonaphthalene, colchicine, or 8-hydroxyquinoline. Subsequently, the root tips of ARs were washed for 30 min in distilled water, fixed overnight in a solution of ethanol and acetic acid (3:1 *v*/*v*), and stored at –20 °C until further use. The root tips were washed in distilled water for 10 min. The root cap was cut and discarded. Then, two to three mm from the root tip was removed, ensuring that it contained the white meristem region. The excised portion was transferred to a tube containing an enzyme cocktail (20 μL for each tip). A cocktail enzyme consisted of cellulase and pectinase. Concentrations of the cocktail enzyme and incubation time for different species were listed in Table S1. Slides were prepared following the method described by Kato et al. [27]. A drop of modified carbol-fuchsin was added to the sample on the slide to stain the chromosomes. Visualization was performed using an Olympus BX41 microscope to assess the quality of metaphase chromosomes. The detailed procedure of chromosome preparations is described in Supplementary Figure S9.

## Figures and Tables

**Table 1:** Plant material.

Type	Genus	Species	Description	Source
Woody plant	*Morus*	*M. alba*	‘Da10’ and ‘Heyebai’	A
	*Broussonetia*	*B*. *papyrifera*	wild material	B
Herbaceous plant	*Lolium*	*L. multiflorum*	‘Nanhei No.1’	A
	*Sorghum*	*S*. *sudanense*	‘Xinsu No.3’	C
		*S. nitidum*	wild material	D
		*S*. *propinquum*	wild material	E
		*S. bicolor×* *S. sudanense*	‘Jicao No.2’	F
	*Zea*	*Z. mays*	‘Yayu No.8’	G
		*Z. mexicana*	‘You 12’	H
	*Glycine*	*G. max*	‘Gongxiadou 12’	I
	*Medicago*	*M. sativa*	‘Sardi’	J
	*Brassica*	*B. napus*	‘Zhongmianyou 783’	K

(A) Sericulture Research Institute, Sichuan Academy of Agricultural Sciences. (B) The wild *Broussonetia papyrifera* was obtained from the Chinese Sericulture Museum located in Shunqing District, Nanchong City, China. (C) College of Grassland Science, Xinjiang Agricultural University. (D) Germplasm Bank of Wild Species in Southwest China. (E) Chongqing Botanical Garden of Medicinal Plants. (F) Hebei Academy of Agriculture and Forestry Sciences. (G) Sichuan Yayu Technology Co. LTP. (H) The seeds were purchased from an E-commerce platform. (I) Zigong Academy of Agricultural Sciences. (J) Barenbrug (Tianjin) International Co. LTD. (K) Nanchong Academy of Agricultural Sciences.

## Data Availability

The supporting dataset for the findings of this article is available in this article along with its Supplementary Materials.

## Supplementary Materials

The following supporting information can be downloaded at https://www.mdpi.com/article/10.3390/ijms252111723/s1.
