# Supplementary material for: Developing Adventitious Root Meristems Induced by Layering for Plant Chromosome Preparation
Source: Int J Mol Sci. 2024 Oct 31;25(21):11723. doi: 10.3390/ijms252111723 (PMC11547123; doi:10.3390/ijms252111723)
Supplement: Supplementary file 1 [file ijms-25-11723-s001.zip › ijms-3290897-supplementary.pdf]

Supplementary Information for

Developing Adventitious Root Meristems Induced by Layering for Plant Chromosome Preparation

Xu Yan [1,2,†,*], Zizhou Wu [1,2,†], Honglin Wang [1,2], Yanchun Zuo [1,2] and Zhouhe Du [1,2,*]

[1] Institute of Special Economic Animals and Plants, Sichuan Academy of Agricultural Sciences, Nanchong 637000, China; wuzizhou@scsaas.cn (Z.W.); hlwang@scsaas.cn (H.W.); zuoyanchun@scsaas.cn (Y.Z.)

[2] Forage Crops Germplasm Innovation and Production Management Key Laboratory of Nanchong City, Sericulture Research Institute, Sichuan Academy of Agricultural Sciences, Nanchong 637000, China

* Correspondence: yanxu@scsaas.cn (X.Y.); duzhouhe@scsaas.cn (Z.D.)

† These authors contributed equally to this work.

This PDF file includes:

Figures S1 to S9
Table S1
SI References

Figures S1 to S9

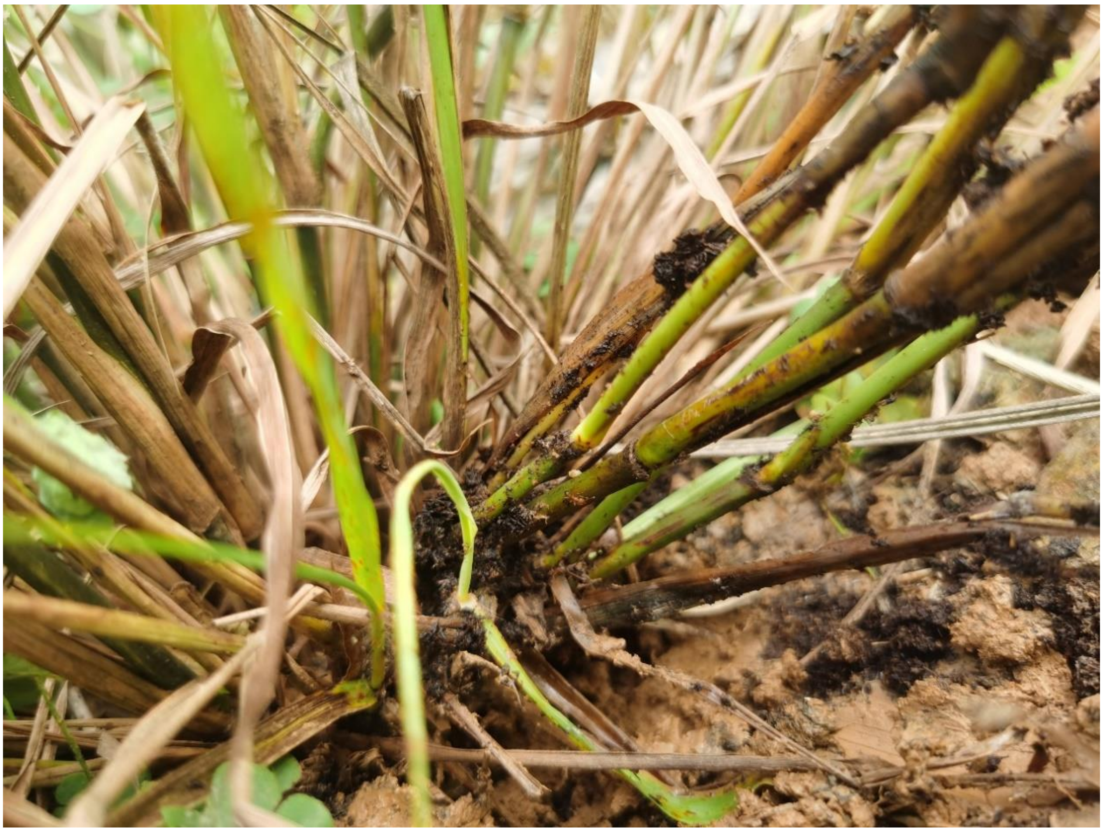

Figure S1. No ARs formation at the stem nodes of one-year-old *Sorghum nitidum*.

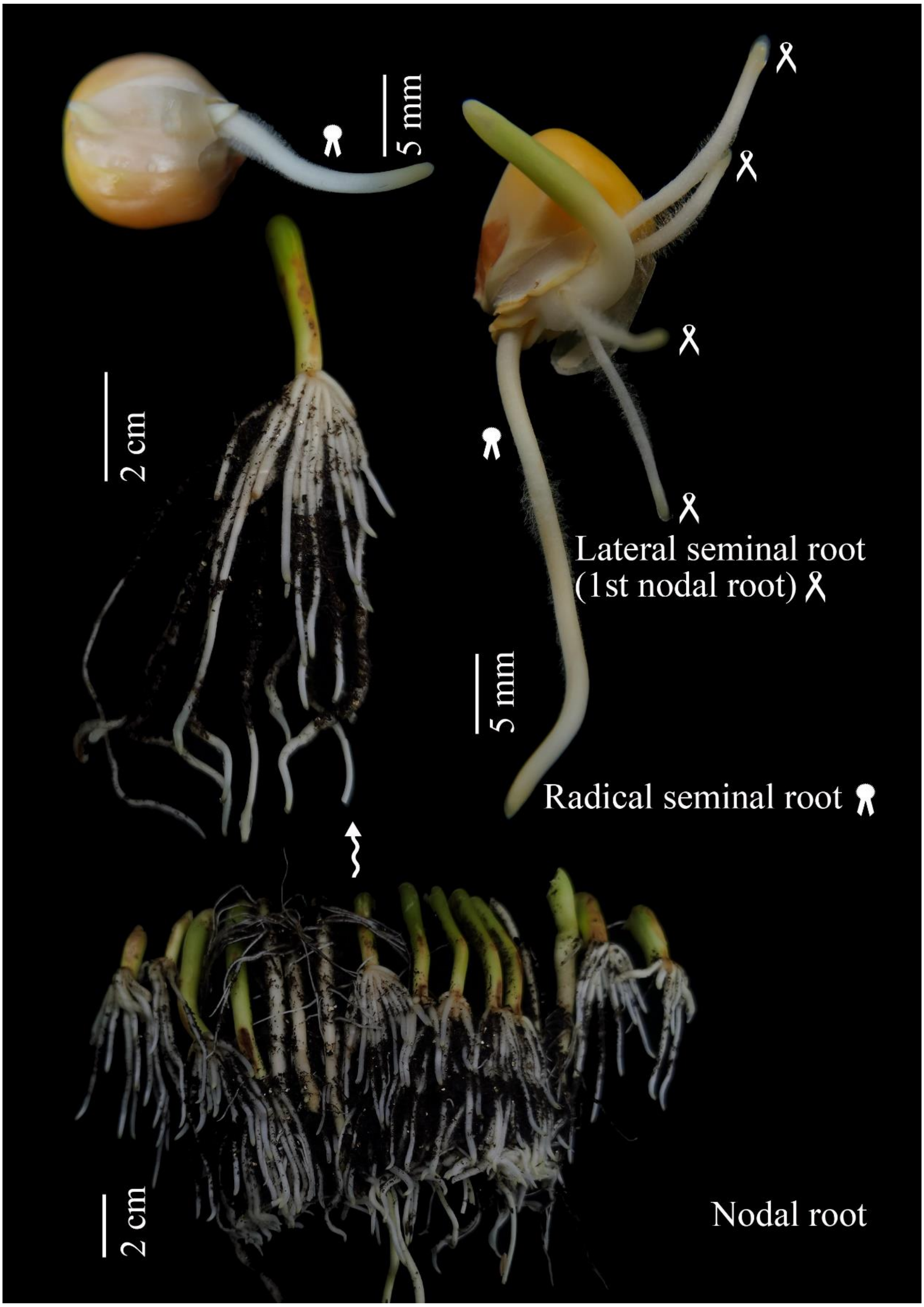

Figure S2. Wound-induced ARs formation at the end of the maize crown roots.

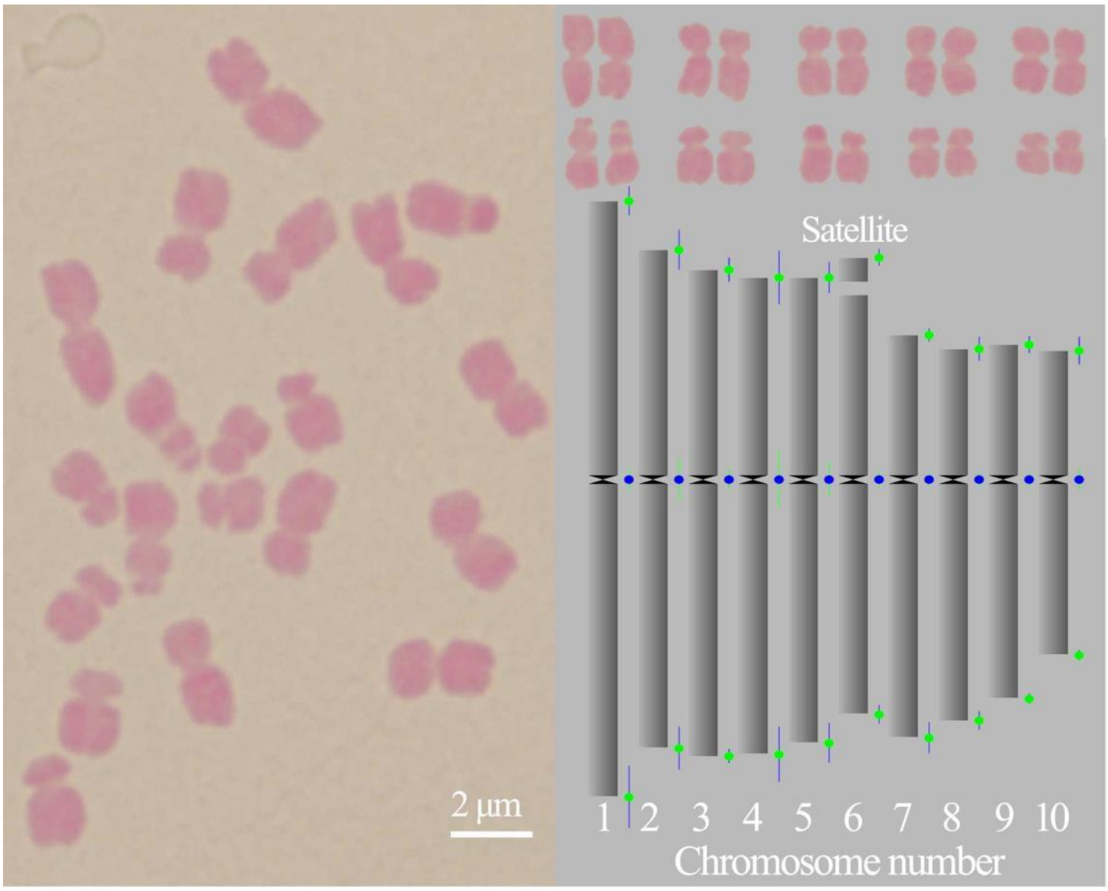

Figure S3. An example of karyotype derived from the chromosomes of maize ARs. Karyotype measurements and idiogram construction were performed by DRAWID (Kirov et al., 2017).

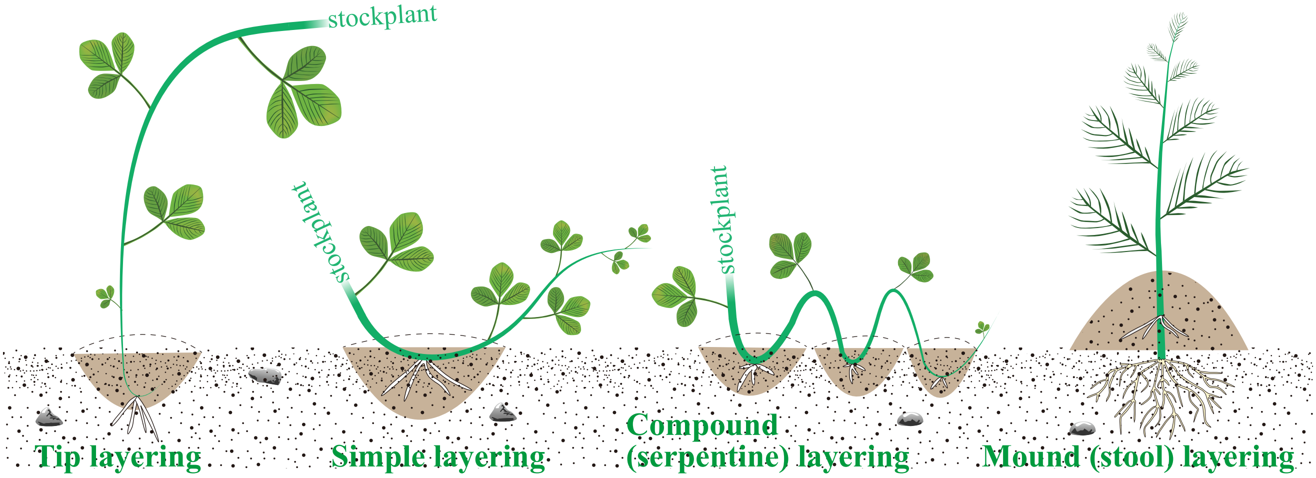

e.g.	e.g.	e.g.	e.g.
dewberry<br>loganberry<br>black raspberry<br>purple raspberry<br>trailing blackberry<br>Other *Rubus* L.	abelia<br>azalea<br>jasmine<br>forsythia<br>boxwood<br>pyracantha<br>wax myrtle<br>honeysuckle<br>climbing rose<br>rhododendron	grapes<br>pothos<br>willow<br>wisteria<br>clematis<br>dogwood<br>viburnum<br>rambler rose<br>vining honeysuckle<br>heart-leaf philodendron	spirea<br>quince<br>daphne<br>magnolia<br>gooseberry<br>cotoneaster<br>apple rootstock

Figure S4. ARs meristems induced by layering.

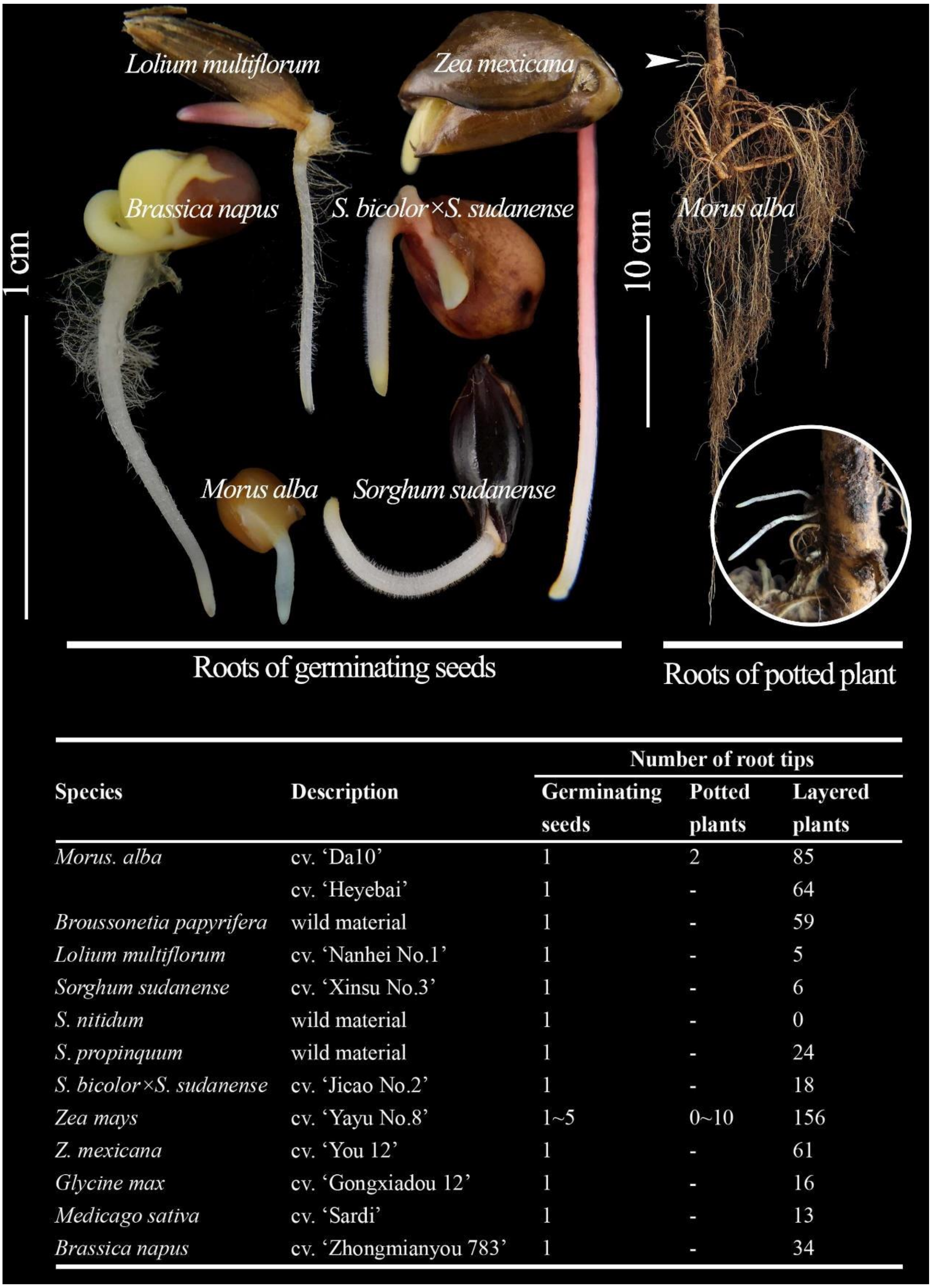

Species	Description	Number of root tips		
		Germinating seeds	Potted plants	Layered plants
Morus. alba	cv. 'Da10'	1	2	85
	cv. 'Heyebai'	1	-	64
Broussonetia papyrifera	wild material	1	-	59
Lolium multiflorum	cv. 'Nanhei No.1'	1	-	5
Sorghum sudanense	cv. 'Xinsu No.3'	1	-	6
S. nitidum	wild material	1	-	0
S. propinquum	wild material	1	-	24
S. bicolor×S. sudanense	cv. 'Jicao No.2'	1	-	18
Zea mays	cv. 'Yayu No.8'	1~5	0~10	156
Z. mexicana	cv. 'You 12'	1	-	61
Glycine max	cv. 'Gongxiadou 12'	1	-	16
Medicago sativa	cv. 'Sardi'	1	-	13
Brassica napus	cv. 'Zhongmianyou 783'	1	-	34

Figure S5. Number of root tips obtained by three types of methods.

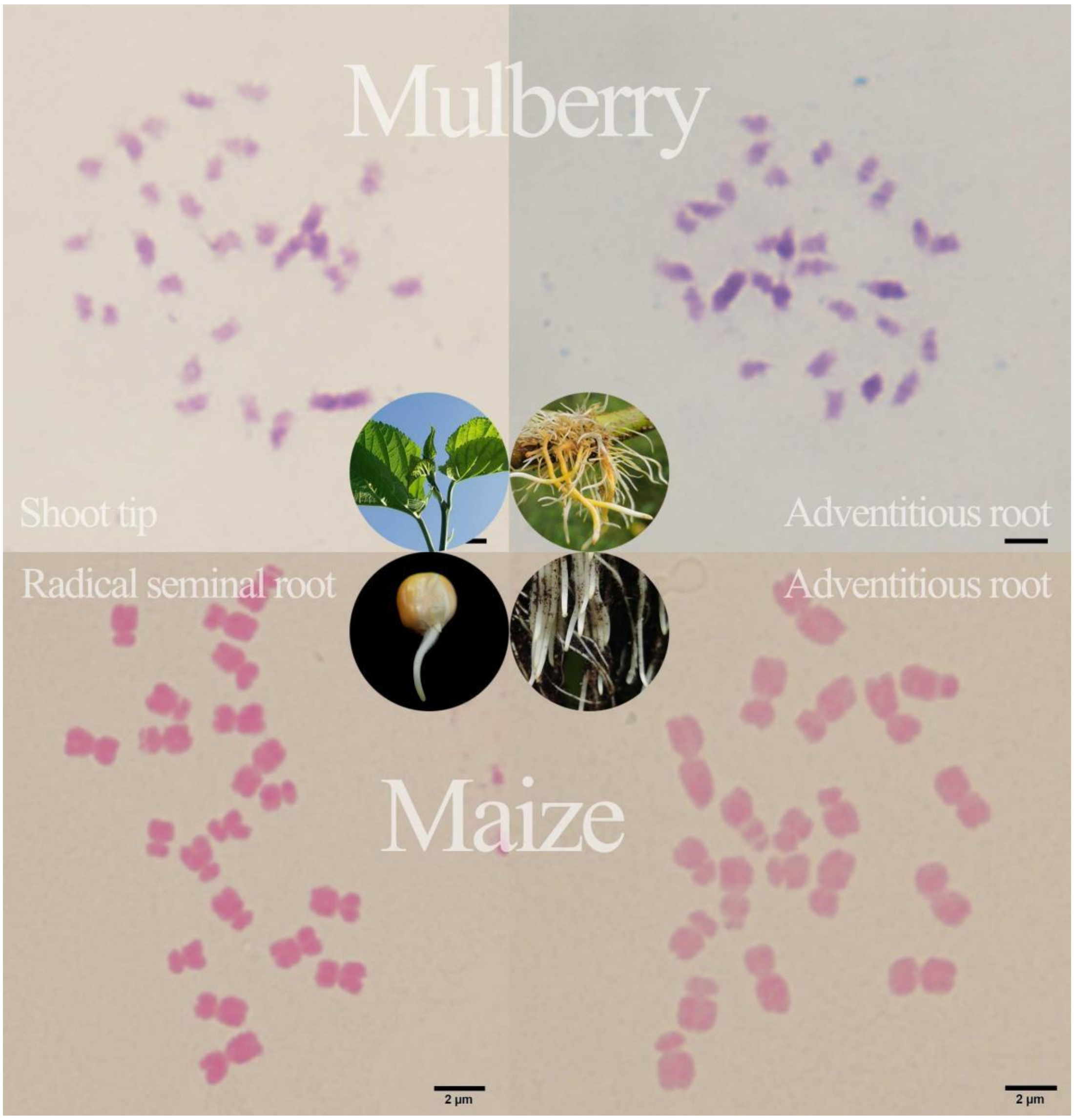

Figure S6. Morphological characteristics of chromosomes from different sources of meristem tissues in mulberry and maize.

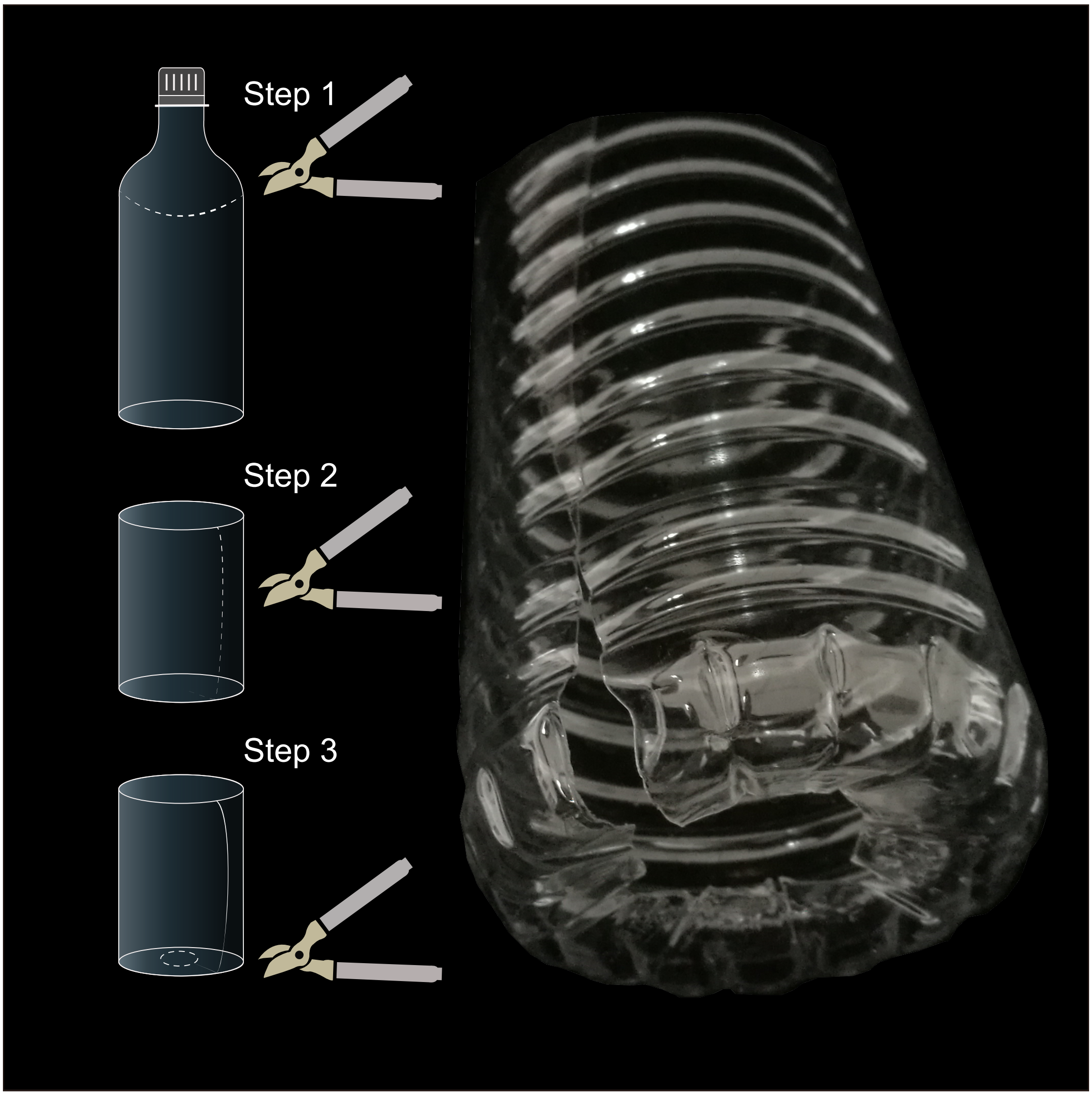

Figure S7. Homemade box for layering.

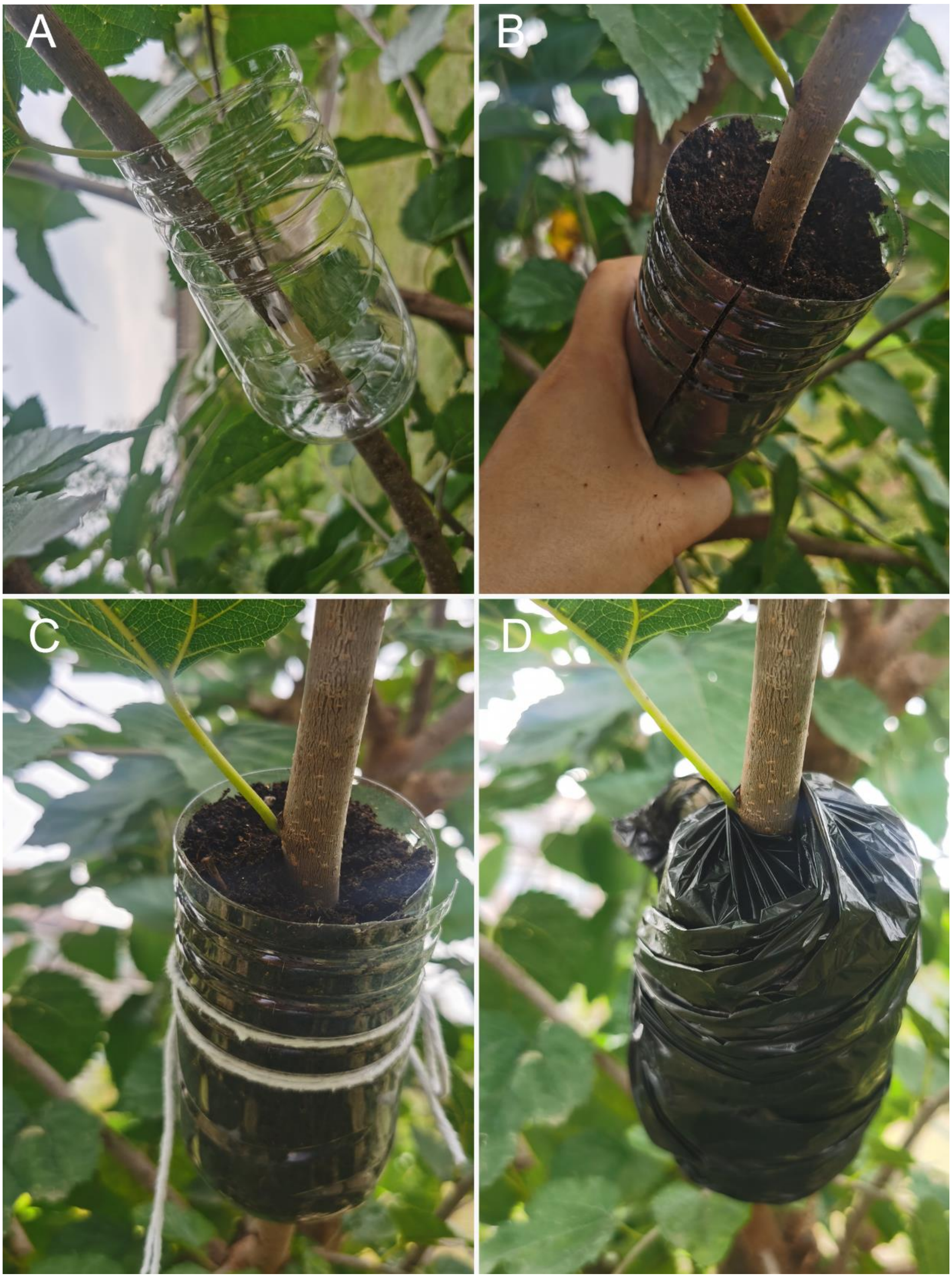

Figure S8. Method for ARs induction in woody plant. (**A**) Make 2 parallel cuts through the bark, peel the ring of bark, scrape the exposed wood, and put on the box. (**B**) Fill box with moist PINDSTRUP moss. (**C**) Fix box with a string. (**D**) Cover box with a black plastic wrap or aluminum foil.

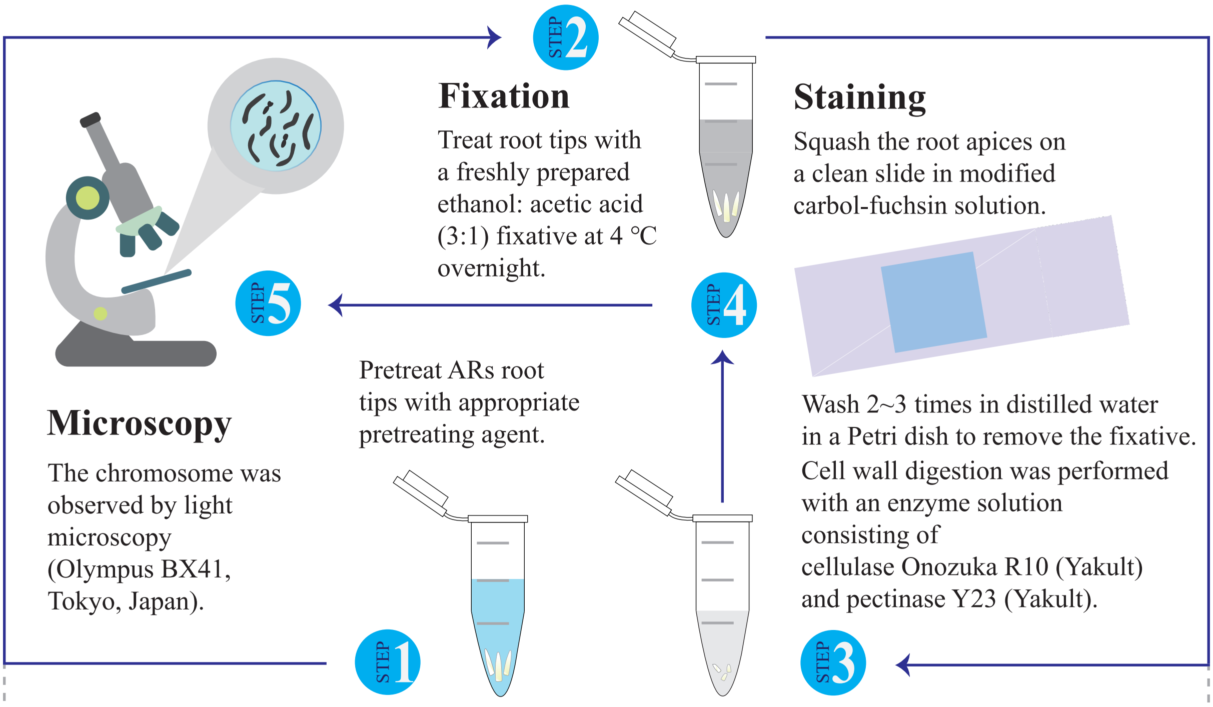

Species	Pretreatment	Enzyme digestion
Morus alba	0.002 mol/L 8-hydroxyquinoline for 2.5 hr at 25 °C	6% (w/v) R10 and 1% (w/v) Y23 (1:1) for 1 hr at 37 °C
Broussonetia papyrifera	A mixture of 0.002 mol/L 8-hydroxy- -quinoline and 0.1% colchicine (1:1) for 6 hr at 4 °C	6% (w/v) R10 and 1% (w/v) Y23 (1:1) for 2 hr at 37 °C
Lolium multiflorum	0.002 mol/L 8-hydroxyquinoline for 3 hr at 25 °C	6% (w/v) R10 and 1% (w/v) Y23 (1:1) for 40 min at 37 °C
Sorghum sudanense	α-bromonaphthalene saturated water for 3 hr at 25 °C	2% (w/v) R10 and 2% (w/v) Y23 (1:1) for 2.5 hr at 28 °C
S. propinqu	α-bromonaphthalene saturated water for 3 hr at 25 °C	2% (w/v) R10 and 2% (w/v) Y23 (1:1) for 2.5 hr at 28 °C
S. bicolor × S. sudanen	α-bromonaphthalene saturated water for 3 hr at 25 °C	2% (w/v) R10 and 2% (w/v) Y23 (1:1) for 2.5 hr at 28 °C
Zea mays	α-bromonaphthalene saturated water for 3 hr at 25 °C	6% (w/v) R10 and 1% (w/v) Y23 (1:1) for 1.5 hr at 37 °C
Z. mexica	α-bromonaphthalene saturated water for 2 hr at 4 °C	6% (w/v) R10 and 1% (w/v) Y23 (1:1) for 1 hr at 37 °C
Glycine max	0.002 mol/L 8-hydroxyquinoline for 2~3 hr at 25 °C	6% (w/v) R10 and 1% (w/v) Y23 (1:1) for 50 min at 37 °C
Medicago sativa	A mixture of 0.002 mol/L 8-hydroxy- -quinoline and 0.1% colchicine (1:1) for 3.5 hr at 25 °C	6% (w/v) R10 and 1% (w/v) Y23 (1:1) for 1.5 hr at 37 °C
Brassica napus	α-bromonaphthalene saturated water for 3 hr at 25 °C	5% (w/v) R10 and 5% (w/v) Y23 (1:1) for 30 min at 25 °C

Figure S9. Chromosome preparation diagram.

Table S1

Table S1. Examples of plants that have been air-layered.

No.	Family	Species name	Common name	References
1	Anacardiaceae	*Anacardium Occidentale*	Cashewnut	Northwood, 1964; Elouaflin et al., 2023
2	Anacardiaceae	*Mangifera indica*	Mango	Singh, 1953; Baghel, 1999
3	Anacardiaceae	*Spondias pinnata*	Hog-plum	Tomar, 2006
4	Annonaceae	*Annona muricata*	Soursop	Chacko et al., 2023
5	Annonaceae	*Annona reticulata*	Custard apple	Chovatia and Singh, 2000; Hazarika et al., 2021
6	Apocynaceae	*Carissa carandas*	Karaunda	Misra and Jaiswal, 1994; Raut et al., 2015
7	Araliaceae	*Schefflera arboricola*	Dwarf umbrella tree	Broschat and Donselman, 1983
8	Asparagaceae	*Dracaena marginata*	Dracaena	Broschat and Donselman, 1983
9	Bombacaceae	*Bombax ceiba*	Semul	Venkatesh et al., 1978
10	Boraginaceae	*Cordia dichotoma*	Lasoda	Chovatia and Singh, 2000
11	Burseraceae	*Canarium schweinfurthii*	African elemi	Kanmegne et al., 2022
12	Caesalpiniaceae	*Hymenaea courbaril*	Jotoba	Thirunavoukkarasu et al., 2004
13	Caesalpiniaceae	*Tamarindus indica*	Tamarind	Ferreira et al., 2017
14	Caesalpinoideae	*Intsia bijuga*	Kwila	Eganathan et al., 2000
15	Clusiaceae	*Allanblackia spp.*	Vegetable tallow tree	Ofori et al., 2015
16	Cupressaceae	*Taxodium distichum*	Bald cypress	Khosla et al., 1979
17	Dipterocarpaceae	*Dipterocarpus turbinatus*	Garjan	Rashid and Serajuddoula, 1984
18	Ebenaceae	*Diospyros ebenum*	Ceylon ebony	Kumar et al., 2019
19	Elaeocarpaceae	*Elaeocarpus angustifolius*	Blue marble tree	Verma et al., 2016
20	Eleagnaceae	*Hippophae*	Seabuckthorn	Chandola et al., 2022

		Salicifolia		
21	Ericaceae	*Macleania rupestris*	Uva camarona	Durán-Casas et al., 2013
22	Ericaceae	*Vaccinium meridionale*	Andean berry	Ligarreto-Moreno et al., 2013
23	Euphorbiaceae	*Excoecaria agallocha*	Milky mangrove	Eganathan et al., 2000
24	Euphorbiaceae	*Lasiococca comberi*		Kamila and Panda, 2019
25	Fagaceae	*Quercus incana*	Oak	Khosla et al., 1979
26	Gentianaceae	*Fagraea auriculata*	Pelir musang	Yeo et al., 2011
27	Irvingiaceae	*Irvingia gabonensis*	African mango	Tchoundjeu et al., 2010
28	Juglandaceae	*Carya illinoensis*	Pecan	Sparks and Chapman, 1970
29	Labiatae	*Gmelina arborea*	Beechwood	Arya and Haque, 1982
30	Lamiaceae	*Vitex madiensis*	Rough fingerleaf	Mapongmetsem and Diksia, 2014
31	Lauraceae	*Cinnamomum aromaticum*	Cassia cinnamon	Krishnamoorthy and Rema, 1994
32	Lauraceae	*Cinnamomum verum*	Ceylon cinnamon	Verma et al., 2013
33	Lauraceae	*Cinnamomum verum*	Cinnamon	Waman and Bohra, 2018
34	Lauraceae	*Eusideroxylon zwageri*	Ironwood	Irawan et al., 2019
35	Lauraceae	*Persea americana*	Avocado	Oliveira et al., 2008
36	Lauraceae	*Persea bombycina*	Som	Ram et al., 2011
37	Lauraceae	*Phoebe cooperiana*	Tapil	Pabin et al., 2021
38	Leguminosae	*Acacia nilotica*	Gum arabic tree	Sharma et al., 2004
39	Leguminosae	*Dalbergia sissoo*	Indian rosewood	Puri and Nagpal, 1988
40	Leguminosae	*Inga feuillei*	Pacay	Brennan and Mudge, 1998
41	Leguminosae	*Parkia biglobosa*	African locust bean	Mapongmetsem and Diksia, 2014
42	Leguminosae	*Parkia speciosa*	Sato	Chanthadet, 1991

43	Leguminosae	*Parkia roxburghii*	Tree bean	Borpuzari et al., 2020
44	Leguminosae	*Prosopis africana*	African mesquite	Abdou et al., 2015
45	Leguminosae	*Prosopis cineraria*	Sponge tree	Solanki et al., 1984
46	Leguminosae	*Prosopis laevigata*	Mesquite	Ramírez-Malagón, et al., 2014
47	Lythraceae	*Punica granatum*	Pomegranate	Munde et al., 2016
48	Malpighiaceae	*Byrsonima crassifolia*	Nance	Duarte and Escobar, 2004
49	Malvaceae	*Grewia optiva*		Nagpal and Sehgal, 1985
50	Malvaceae	*Heritiera fomes*	Sunder	Eganathan et al., 2000
51	Malvaceae	*Scaphium macropodum*	Malva nut tree	Nache Gowda et al., 2006
52	Meliaceae	*Azadirachta indica*	Thai neem	Kumar et al., 2002
53	Meliaceae	*Dysoxylum malabaricum*		Hussain et al., 2013
54	Meliaceae	*Khaya anthotheca*	White mahogany	Filho et al., 2016
55	Meliaceae	*Xylocarpus granatum*	Cannonball mangrove	Kathiresan and Ravikumar, 1995
56	Moraceae	*Acacia catechu*	Khair tree	Puri and Nagpal, 1988
57	Moraceae	*Atrocarpus altilis*	Breadfruit	Bridgemohan et al., 2016
58	Moraceae	*Artocarpus heterophyllus*	Jackfruit	Alila et al., 2000
59	Moraceae	*Ficus auriculata*	Roxburgh fig	Tomar and Singh, 2011
60	Moraceae	*Ficus benjamina*	Weeping fig	Broschat and Donselman, 1983
61	Moraceae	*Ficus carica*	Fig	Puri and Nagpal, 1988; Reddy et al., 2014
62	Moraceae	*Ficus elastica*	Rubber plant	Broschat and Donselman, 1983
63	Moraceae	*Ficus krishnae*		Tomar and Singh, 2011
64	Moraceae	*Morus alba*	Mulberry	Puri and Nagpal, 1988
65	Myristicaceae	*Myristica fragrans*	Nutmeg	Nissar et al., 2019
66	Myrtaceae	*Campomanesia phaea*	Cambuci	Santoro, et al., 2021
67	Myrtaceae	*Eucalyptus*		Husain and

		microtheca		Ponnuswamy, 1964
68	Myrtaceae	*Plinia trunciflora*	Jabuticaba	Danner et al., 2006
69	Myrtaceae	*Psidium guajava*	Guava	Baghel et al., 2016; Verma et al., 2019
70	Myrtaceae	*Syzygium jambos*		Mirihagalla and Fernando, 2020
71	Myrtaceae	*Syzygium grande*	Dhakijam	Rashid and Serajuddoula, 1984
72	Myrtaceae	*Syzygium guineense*	Woodland waterberry	Mapongmetsem and Diksia, 2014
73	Myrtaceae	*Syzygium javanica*	Waterapple	Pau and Aditi, 2009
74	Myrtaceae	*Syzygium samarangense*	Wax apple	Khandakera et al., 2022
75	Oleaceae	*Olea europaea*	Olive	Rehman, 2013
76	Phyllanthaceae	*Uapaca kirkiana*	Wild loquat	Mwang'ingo and Lulandala, 2011
77	Pinaceae	*Pinus massoniana*	Pine	Khosla et al., 1979
78	Pinaceae	*Pinus radiata*	Pine	Khosla et al., 1979
79	Pinaceae	*Pinus roxburghii*	Pine	Khosla et al., 1979
80	Poaceae	*Guadua angustifolia*	Bamboo	Verma et al., 2013
81	Ranunculaceae	*Paeonia suffruticosa*	Peony	Zhang et al., 2022
82	Rosaceae	*Chaenomelis sinensis*	Japonês quince	Pio et al., 2007
83	Rosaceae	*Prunus azorica*	Azorean cherry	Moreira et al., 2009
84	Rosaceae	*Prunus persica*	Peach	Castro and Silveira, 2003
85	Rosaceae	*Rosa hybrida*	Rose	Dartey et al., 2002
86	Rosaceae	*Prunus domestica*	Plum	Mozumder et al., 2017
87	Rubiaceae	*Gardenia gummifera*	Gummy gardenia	Sharda and Verma, 2010
88	Rubiaceae	*Lophira lanceolata*	Dwarf red ironwood	Mapongmetsem and Diksia, 2014
89	Rubiaceae	*Neolamarckia cadamba*	Kadamb	Singh, 2013
90	Rutaceae	*Citrus aurantifolia*	Acid lime/ Kagzilime	Verma et al., 2022; Mishra, 2014
91	Rutaceae	*Citrus hystrix*	Magrood	Farah et al., 2009

92	Rutaceae	*Citrus limon*	Lemon	Lalramhluna and Prasad, 2016
93	Rutaceae	*Citrus sinensis*	Sweet orange	Dutta, 2000
94	Rutaceae	*Zanthoxylum armatum*	Nepal pepper	Purohit et al., 2016
95	Santalaceae	*Osyris lanceolata*	African sandalwood	Mwang'ingo et al., 2006
96	Sapindaceae	*Blighia sapida*	Jamaican ackee	Maurya et al., 2013
97	Sapindaceae	*Litchi chinensis*	Lychee	Janos et al., 2001
98	Sapindaceae	*Nephelium lappaceum*	Rambutan	Bhattacharjee et al., 2018
99	Sapotaceae	*Synsepalum dulcificum*	Miracle fruit	Geoffery and Sani, 2017
100	Sapotaceae	*Vitellaria paradoxa*	Shea tree	Yeboah et al., 2014
101	Simmondsiaceae	*Simmondsia chinensis*	Jojoba	Bashir et al., 2005
102	Sonneratiaceae	*Sonneratia apetala*		Kathiresan and Ravikumar, 1995
103	Theaceae	*Camellia sinensis*	Tea	Widyastuti et al., 2020
104	Ximeniaceae	*Ximenia americana*	Tallow wood	Mapongmetsem and Diksia, 2014

SI References

Abdou, L., Karim, S., Habou, R., and Mahamane, A. (2015). Vegetative propagation trial of *Prosopis africana* (Guill. et Perr.) Taub. by air layering under Sudano-Sahelian climate in the South-Central Niger. J. Bot. 2015:286582.

Alila, P., Chiesotsu, S., Kar, P.L., and Sanyal, D. (2000). Influence of growth regulators and etiolation on air layering in jackfruit. Hortic. J. 13:9–13.

Arya, R.S., and Haque, M.S. (1982). Grafting and budding in Yemane *Gmelina arborea*. Roxb. Indian For. 108:497–500.

Baghel, B.S. (1999). Response of air layering of mango to coloured polywrappers. Indian J. Hortic. 56:133–134.

Baghel, M., Raut, U.A., and Ramteke, V. (2016). Effect of IBA concentrations and time of air-layering in guava cv. L-49. Res. J. Agric. Sci. 7:117–120.

Bashir, M.A., Ahmad, M., and Anjum, M.A. (2005). Response of six promising jojoba strains to air layering. Biosci. Res. 3:172–177.

Bhattacharjee, P., Sakthivel, T., Naik, N., Gowda, IN.D., Aswath, C., Nataraja, K.H., Awachare, C., and Kumar, R.S. (2018). Effects of rooting media and different IBA concentrations on air layering of Rambutan (*Nephelium lappaceum* L.). 6:3300–3304.

Borpuzari, P.P., Singh, M.K., and Dutta, A. (2020). Air layering of *Parkia roxburghii* G. Don Syn. P. timoriana (DC.) Merr. A high value tree species of northeast east India. Pharma Innovation J. 9:192–194.

Brennan, E.B., and Mudge, K.W. (1998). Vegetative propagation of *Inga feuillei* from shoot cuttings and air layering. New For. 15:37–51.

Bridgemohan, P., Mohamed, M.El.S., Ramoutar, A., Singh, K., and Bridgemohan, R. (2016). Air layering (Marcotting) of breadfruit (*Artocarpus altilis*). Int. J. Res. Sci. Innovation 3:1–4.

Castro, L.A.S., and Silveira, C.A.P. (2003). Vegetative propagation of peach by air layering technique. Rev. Bras. Frutic. 25:368–370.

Chacko, I., Ranchana, P., Gopi, V., Srinivasan, J., Dinesh Kumar P., Bharanidharan A., Pavethra A., Vishal S., Giriprasath R.S., and Savitha V. (2023). Effect of time and IBA concentrations on Soursop (*Annona muricata*): Air layering. Pharma Innovation J. 12:815–818.

Chandola, J., Chamola, B.P., and Dhiman, M. (2022). Propagation of *Hippophae*

salicifolia D.Don through air-layering. J. Mountain Res. 17:327–331.

Chanthadet, S. (1991). Asexual propagation methods of sato (*Parkia speciosa* Hassk) National AGRIS Centre, Kasetsart University, Thailand, Prince of Songkla Univ. Pattani Campus, Pattani (Thailand). Faculty of Science and Technology. Dept. of Science and Mathematics.

Chovatia, R.S., and Singh, S.P. (2000). Effect of ringing of shoots and treatment with IBA and NAA on success of air-layering in lasoda (*Cordia dichotoma* Forst.). Adv. For. Res. India 22:173–181.

Chovatia, R.S., and Singh, S.P. (2000). Success of air-layering in custard apple (*Annona squamosa* L.) as influenced by ringing of shoots and growth regulators. Orissa J. Hortic. 28:61–65.

Danner, M.A., Citadin, I., Junior, A.D.A.F., Assmann, A.P., Mazaro, S.M., Donazzolo, J., and Sasso, S.A.Z. (2006). Taking roots of 'jabuticaba' fruit tree (*Plinia trunciflora*) by air layering technique. Rev. Bras. Frutic. 28:530–532.

Dartey, P.K.A., Adzraku, H., Atuah, L., Boateng, P.Y., and Awotwe, J. (2002). Use of a polymeric super-absorbent in air layering of roses (*Rosa hybrida*). J. Sci. Technol. 22:799.

Duarte, O., and Escobar, O. (2004). Propagation of nance (*Byrsonima crassifolia* (L) H.B.K.) by air layering and grafting. Proceedings of the Interamerican Society for Tropical Horticulture 47:170–171.

Durán-Casas, S., Veloza-Suan, C., Magnitskiy, S., and Lancheros, H.O. (2013). Evaluation of uva camarona (*Macleania rupestris* Kunth A.C. Smith) propagation with air layering. Agron. Colomb. 31:18–26.

Dutta, P. (2000). Effect of growth regulators on air-layering of sweet orange *Citrus sinensis* (L.) Osbeck. Environ. Ecol. 18:899–901.

Eganathan, P., Rao, C.S., and Anand, A. (2000). Vegetative propagation of three mangrove tree species by cuttings and air layering. Wetlands Ecol. Manage. 8:281–286.

Elouaflin, A.Y.A., Kouakou, K.L., Kouakou, C., Dao, J.P., and Zoro, B.I.A. (2023). Branch diameters, substrates, and indole-3-butyric acid effect on cashew (*Anacardium occidentale* L.) tree propagation by air layering. Forestist 73:278–284.

Farah Fazwa, M.A., Ab Rasip, A.G., and Lokmal, N. (2009). The effect of growing media on rooting ability in air layering propagation of *Citrus hystrix*.

J. Trop. Med. Plants 10:101–104.

Ferreira, A.F.A., Boliani, A.C., Monteiro, L.N.H., da Silva, M.S.C., Rodrigues, M.G.F., Faria, G.A., Pigari, L.B., Lopes, B.G., Felizardo, L.M., and Peixoto, A.P.B. (2017). Substrates and indolebutyric acid (IBA) concentrations in air-layering rooting of Tamarind tree. Afr. J. Agric. Res. 12:2926–2932.

Filho, J.B., Carvalho, M.A.D., de Oliveira, L.S., Konzen, E.R., Campos, W.F., and Brondani, G.E. (2016). Propagation of *Khaya anthotheca*: interspecific grafting with swietenia macrophylla and air layering. CERNE 22:475–484.

Geoffery, R.M., and Sani, H. (2017). Promotion of adventitious root formation of miracle fruit (*Synsepalum dulcificum* Daniell) through stem cuttings and air layering technique. Trans. Sci. Technol. 4:1–7.

Gohil, J.H. (2014). Effect of different concentrations of IBA and NAA on air layering of cashewnut cv. Vengurla 4. Trends Biosci. 7:351–354.

Hazarika, J., Hazarika, D.N., and Langthasa S. (2021). Standardization of propagation method of custard apple by air Layering. J. AgriSearch 8:222–228.

Husain, A.M.M., and Ponnuswamy, P.K. (1964). Preliminary observations on air-layering in *Eucalyptus microtheca*. Indian For. 90:484–487.

Hussain, A., Pandurangan, A.G., and Remya, R. (2013). Clonal propagation through stem cuttings and air layering in *Dysoxylum malabaricum* Bedd. ex Hiern. - an endemic and rare tree species of the Western Ghats. Indian J. For. 36:187–190.

Irawan, B., Tamin, R.P., and Hardiyanti, R.A. (2019). Effects of indole acetic acid (IAA) Indole butyric acid (IBA) to the growth and rooting of ironwood (*Eusideroxylon zwageri* Teijsm. & Binn.) air layering. J. Man. Hut. Trop. 25:126–134.

Janos, D.P., Schroeder M.S., Schaffer, B., and Crane, J.H. (2001). Inoculation with arbuscular mycorrhizal fungi enhances growth of *Litchi chinensis* Sonn. trees after propagation by air-layering. Plant Soil 233:85–94.

Kamila, P.K., and Panda, P.C. (2019). Large-scale vegetative propagation of *Lasiococca comberi* by air layering. J. Trop. For. Sci. 31:37–42.

Kanmegne, G., Atchioutchoua, R.S., and Noumbo, G.R.T. (2022). Combining air-layering and stem cutting techniques to optimize the production of quality planting materials for agroforestry tree species: a case study of

Canarium schweinfurthii Engl. (Burseraceae), For. Trees Livelihoods 31:170–183.

Kathiresan, K., and Ravikumar, S. (1995). Vegetative propagation through air-layering in two species of mangroves. Aquat. Bot. 50:107–110.

Khandaker, M.M., Saidi, A., Badaluddin, N.A., Yusoff, N., Majrashi, A., Alenazi, M.M., Saifuddin, M., Alam, Md.A., and Mohd, K.S. (2022). Effects of indole-3-butyric acid (IBA) and rooting media on rooting and survival of air layered wax apple (*Syzygium samarangense*) cv Jambu Madu. Braz. J. Biol. 82:e256277.

Khosla, P.K., Chauhan, P.S., and Sood, R. (1979). Air-layering studies in some forest trees. Indian J. For. 2:161–164.

Kirov, I., Khrustaleva, L., Laere, K.V., Soloviev, A., Meeus, S., Romanov, D., and Fesenko, I. (2017). DRAWID: user-friendly java software for chromosome measurements and idiogram drawing. Comp. Cytogen. 11:747–757.

Krishnamoorthy, B., and Rema, J. (1994). Air layering in cassia (*Cinnamomum aromaticum* Nees.). J. Spices Aromat. Crops 3:48–49.

Kumar, R.V., Gupta, V.K., Ahlawat, S.P., and Datta, A. (2002). Vegetative propagation through air layering in Thai neem (*Azadirachta indica* var siamensis Valenton). Indian J. Agroforestry 4:135–137.

Kumar, V., Maheswarappa, V., Hegde, R., and Salimath, S.K. (2019). Vegetative propagation through air layering of *Diospyros ebenum* J. Koenig: An endangered tree species. Int. J. Curr. Microbiol. Appl. Sci. 8:1568–1574.

Lalramhluna, P., and Prasad, V.M. (2016). Effect of different levels of indole-3-butyric acid on growth, development, survival and establishment of air layered lemon (*Citrus limon* L. Burm.) cv. assam lemon under Allahabad agro-climatic condition. Int. J. Life. Sci. Scienti. Res. 2:599–603.

Ligarreto-Moreno, G.A., Torres-Aponte, W.S., and Ariza-Castillo, C.A. (2013). Propagation of the neotropical fruit *Vaccinium meridionale* Swartz by air layering. Agron. Colomb. 31:169–175.

Mapongmetsem, P.M., and Diksia, M. (2014). Vegetative propagation of local fruit trees by air layering in the Guinean savannah highlands (GSH). J. Sustainable For. 33:21–32.

Maurya, R.P., Lewis, D.M., and Chandler, J.St.A. (2013). Studies on the propagation of Jamaican ackee (*Blighia sapida* L.) by air-layering. Hortscience 48:1298–1300.

Mirihagalla, M.K.P.N., and Fernando, K.M.C. (2020). Effect of *Aloe vera* gel for inducing rooting of stem cuttings and air layering of plants. J. Dry Zone Agric. 6:13–26.

Mishra, S. (2014). Effect of different rooting media on survival and success of air layers in kagzilime. Ann. Plant Soil Res. 16:264–267.

Misra, K.K., and Jaiswal, H.R. (1994). Effect of growth regulators on rooting and survival of air layers of karaunda (*Carissa carandas* L.). Ann. Agric. Res. 11:208–210.

Moreira, O., Martins, J., Silva, L., and Moura, M. (2009). Propagation of the endangered Azorean cherry *Prunus azorica* using stem cuttings and air layering. Arquipélago Life Mar. Sci. 26:9–14.

Mozumder, S.N., Haque, M.I., Ara, R., Sarker, D., and Shahiduzzaman, M. (2017). Effect of air layering time and genotype on success of plum propagation. Int. J. Biol. Sci. 4:55–61.

Munde, G.R., Nainwad, R.V., Maske, S.N., and Pawar, J.V. (2016). Effect of IBA and other chemicals on air layering in pomegranate cv. Bhagwa. Bioinfolet 13:291–293.

Mwang'ingo, P.L.P., and Lulandala L.L. (2011). Air layering and its potential in propagating *Uapaca kirkiana*: A fruit tree from the miombo woodland, Tanzania. South. For. 73:67–71.

Mwang'ingo, P.L., Teklehaimanot, Z., Lulandala, L.L., and Maliondo, S.M. (2006). Propagating *Osyris lanceolata* (African sandalwood) through air layering: Its potential and limitation in Tanzania. South. Afr. For. J. 207:7–13.

Nache Gowda, V., Vasanth, K.R., and Shyamalamma, S. (2006). Studies on vegetative propagation of khirni (*Manilkara hexandra* (Roxb) Dub.) by air layering. Acta Hortic. 727:85–88.

Nagpal, R., and Sehgal, R.N. (1985). Propagation of some agro forestry species by air layering. Indian J. For. 8:161–165.

Nissar, V.A.M., Sasikumar, B., Aarthi, S., and Rema, J. (2019). Air layering in nutmeg (*Myristica fragrans* Houtt.). J. Spices Aromat. Crops 28:66–69.

Northwood, P.J. (1964). Vegetative propagation of cashew (*Anacardium occidentale* L.) by the air-layering method. East Afr. Agric. For. J. 30:35–37.

Ofori, D.A., Asomaning, J.M., Peprah, T., Agyeman, V.K., Anjarwalla, P., Tchoundjeu, Z., Mowo, J.G., and Jamnadass, R. (2015). Addressing

constraints in propagation of *Allanblackia* spp. through seed sectioning and air layering. J. Exp. Biol. Agric. Sci. 3:89–96.

Oliveira, I.V.D.M., Cavalcante, Í.L., Franco, D., and Martins, A.B.G. (2008). Cloning of avocado cultivar "duke 7" (*Persea americana* mill.) using air-layering technique. Rev. Bras. Frutic. 30:759–763.

Pabin, J., Lyngdoh, N., Mehra, T.S., Devi, M.B., and Payum, T. (2021). Effect of phytohormones on rooting behaviour in air layers of *Phoebe cooperiana*, an economically important tree species of Arunachal Pradesh, India. Curr. Bot. 12:192–196.

Paul, R., and Aditi, Ch. (2009). IBA and NAA of 1000 ppm Induce more improved rooting characters in air-layers of waterapple (*Syzygium javanica* L.). Bulg. J. Agric. Sci. 15:123–128.

Pio, R., Dall'Orto, F.A.C., Alvarenga, Â.A., Abrahão, E., Chagas E.A., and Signorini G. (2007). Propagation of Japonês quince for cutting and air layering technique in different periods. Cienc. Agrotecnol. 31:570–574.

Puri, S., and Nagpal, R. (1988). Effects of auxins on air layers of some agroforestry species. Indian J. For. 11:28–32.

Purohit, S., Bhatt, A., Bhatt, I.D., and Nandi, S.K. (2016). Propagation through air layering in *Zanthoxylum armatum* DC: An endangered medicinal plant in the Himalayan region. Proc. Natl. Acad. Sci., India, Sect. B Biol. Sci. 86:607–610.

Ram, R., Samson, M.V., and Bhatt, M.M. (2011). Variation in air-layering response of eight genotypes of *Persea bombycina* Kost. Indian For. 137:1020–1022.

Ramírez-Malagón, R., Delgado-Bernal, E., Borodanenko, A., Pérez-Moreno, L., Barrera-Guerra, J.L., Núñez-Palenius, H.G., and Ochoa-Alejo, N. (2014). Air layering and tiny-air layering techniques for mesquite [*Prosopis laevigata* (H. B. ex Willd.) Johnst. M. C.] tree propagation. Arid Land Res. Manage. 28:118–128.

Rashid, M.H., and Serajuddoula, M. (1984). Vegetative propagation of dhakijam and garjan by air-layering. Bano Biggyan Patrika. 13:64–66.

Raut, U.A., Jadhav, G.G., Bhogave, A.F., and Deshmukh, M.S. (2015). Effect of different IBA levels on air layering of karonda (*Carissa carandas* L.). Res. Crop. 16:537–541.

Reddy, P.P.N., Ray, N.R., Patel, A.D., and Patel, J.S. (2014). Effect of rooting media and IBA (indole butyric acid) levels on rooting and survival of air layering in fig (*Ficus carica* L.) cv. Poona under middle Gujarat agro-climatic conditions. Asian J. Hortic. 9:1–5.

Rehman, M. (2013). Response of olive cultivars to air-layering at various timings. Pak. J. Agric. Res. 50:555–558.

Santoro, M.B., Brogio, B., Bueno, S.C.S., Tanaka, F.A.O., Jacomino, A.P., and Silva, S.R. (2021). Vegetative propagation of *Campomanesia phaea* by the air-layering and grafting techniques. Pesqui. Agropecu. Bras. 56:e02402.

Sharda, A.K., and Verma, S.K. (2010). Air-layering in *Gardenia gummifera* L. F. Indian For. 136:266–268.

Sharma, S.K., Verma, S.K., and Bhojvad, P.P. (2004). Cloning of *Acacia nilotica* for multiplication and establishment of clonal seed orchard. In: Multipurpose trees in the Tropics: Management and Improvement strategies. AFRI, Jodhpur, 691–700.

Singh, L.B. (1953). Vegetative propagation of mango (*Mangifera indica* L.) by air-layering (Gootee). Science 117:158–159.

Singh, S. (2013). Air layering for cloning elite genotypes of Kadamb, (*Neolamarckia cadamba* (Roxb.) Bosser. Indian Botanists Blog-o-Journal. http://www.indianbotanists.com/2013/09/air-layering-for-cloning-elite.html

Solanki, K.R., Kackar, N.L., and Jindal, S.K. (1984). Propagation in *Prosopis cineraria* (L) Mac bride by air-layering. Curr. Sci. 53:1166–1167.

Sparks, D., and Chapman, J.W. (1970). The effect of indole-3-butyric acid on rooting and survival of air-layered branches of the pecan, *Carya illinoensis* Koch, cv. 'Stuart'1. J. Am. Soc. Hortic. Sci. 5:445–446.

Tchoundjeu, Z., Tsobeng, A.C., Asaah, E., and Anegbeh, P. (2010). Domestication of *Irvingia gabonensis* (Aubry Lecomte) by air layering. J. Hortic. For. 2:171–179.

Thirunavoukkarasu, M.B., and Dhal, N.K. (2004). Vegetative propagation of *Hymenaea courbaril* by air layering. J. Trop. For. Sci. 16:268–270.

Tomar, A. (2016). Impact of seasonal changes on air layering and rooting hormone in *Spondias pinnata* (J. Koenig ex L. f.) Kurz. Trop. Plant Res. 3:131–135.

Tomar, A., and Singh, V.R.R. (2011). Effect of air layering time (season) with the

aid of indole butyric acid in *Ficus krishnae* and *Ficus auriculata*. Indian For. 137:1363–1365.

Venkatesh, C.S., Arya, R.S., and Emmanuel, C.J.S.K. (1978). A Note on air layering and budding in semul. Indian For. 104:142–144.

Verma, B., and Sahu, G.D. (2022). Studies on the effect of different rooting media on survival and success of air layering in acid lime (*Citrus aurantifolia* Swingle) under Chhattisgarh plain. Pharma Innovation 11:225–228.

Verma, B., Dhakad, R.K., Bhadauriya, P., Parmar, U., and Tomar, K.S. (2019). A study of different concentration of IBA and NAA on rooting per cent of guawa (*Psidium guajava* L.), air layering. J Pharmacogn. Phytochem. 8:773–775.

Verma, P.K., Das, N., Kaushik, P.K., Kumar, V., and Yadav, A. (2013). Vegetative propagation through air layering of *Guadua angustifolia* Kunth - A commercially important bamboo. Indian For. 139:1088–1091.

Verma, P.K., Das, N., Kumar, V., and Kumar, R. (2013). Effect of *Sphagnum* spp. as substrate media on rooting response of *Cinnamomum verum* presl. (syn. C. zelayanicum Bluma) through air layering. J. Non-Timber For. Prod. 20:179–182.

Verma, P.K., Das, N., Kumar, V., Kaushik, P.K., and Yadav, A. (2016). Air-layering through Sphagnum moss in *Elaeocarpus angustifolius* Blume (Rudraksha)-commercially important tree species. Indian For. 142:875–877.

Waman, A.A., and Pooja, B. (2018). Air layering in *cinnamon* (*Cinnamomum verum* L.) under wet humid tropical conditions. J. Spices Aromat. Crops 27:71–73.

Widyastuti, I.B., Yudono, P., and Putra, E.T.S. (2020). Effects of auxin and cytokinin levels on the success of air layering in tea plant clones of GMB 7 and GMB 9 using husk charcoal, cocopeat and moss media. Indones. J. Agric. Sci. 5:86–91.

Yeboah, J., Banful, B.K.B., Boateng, P.Y., Amoah, F.M., Maalekuu, B.K., and Lowor, S.T. (2014). Rooting response of air-layered shea (*Vitellaria paradoxa*) trees to media and hormonal application under two different climatic conditions. Am. J. Plant Sci. 5:1212–1219.

Yeo, C.K., Ng, B.Y.Q., Ng, P.X., Chong, K.Y., Lok, A.F.S.L., Ang, W.F., Tan, S.Y., and Tan, H.T.W. (2011). Air-layering: a suitable method for mass-

propagating the nationally critically endangered *Fagraea auriculatum* jack (Gentianaceae). Nature Singapore 4:383–392.

Zhang, Y., Yu, S.Y., and Hu, Y.H. (2022). Air layering improves rooting in tree peony cultivars from the Jiangnan group. Horticulturae 8:941.
